# Analysis of the social and psychosocial factors associated with adherence to antiretroviral therapy in adolescents with perinatal HIV-1 infection in Panama from a gender perspective

**DOI:** 10.1080/09540121.2016.1176669

**Published:** 2016-07-08

**Authors:** Dora Estripeaut, Kathia Luciani, Ricardo García, Rita Banús, Trina María Aguais, Edilma Berrío, Alma Jenkins, Sharene Smoot

**Affiliations:** ^a^ Department of Infectious Diseases, Pediatric Hospital “Dr. Jose Renan Esquivel”, Panamá, Republic of Panamá; ^b^ Department of Infectious Diseases, Pediatric Specialties of Social Security Hospital “Dr. Arnulfo Arias”, Panamá, Republic of Panamá; ^c^ ONUSIDA Panama, Panamá, Republic of Panamá; ^d^ AID for AIDS Panama, Panamá, Republic of Panamá; ^e^ UNFPA Panama, Panamá, Republic of Panamá; ^f^ UNICEF Panama, Panamá, Republic of Panamá; ^g^ Independent Statistical Advisor

**Keywords:** Adolescents, retroviral therapy, adherence, social factors, psychosocial factors

## Abstract

Adherence is vital for an effective antiretroviral treatment. This cross-sectional study explored social and psychosocial factors associated with adherence among adolescents with perinatal human immunodeficiency virus type 1 infection in Panama from a gender perspective. A questionnaire developed for the study was applied to 38 adolescent patients (20 female, 18 male; median age, 14 years). Thirty-two patients (86%; one missing response) still depended on an adult to remember taking their medication, among whom 28 relied on a female relative. Although 18 (47%) patients reported to become ill no more than once a year, only 10 (26%) patients showed an undetectable viral load, and 4 (11%) patients showed no CD4 suppression. Seventeen (45%) patients recalled correctly their medication. During the week prior to the interview, 26 patients (68%) reported that they had missed at least one dose. When asked the reason for missing a dose, 23 out of 34 (68%; 4 missing responses) patients responded, “I forgot”. Female patients gave more excuses for missing doses (mean ± SD number of excuses per female, 2.4 ± 2; per male, 1.2 ± 1; *p* = .02), while more male than female patients described an action plan if they ran out of medication (13 vs. 8; *p* = .05). Educational programs involving patients and also family members are warranted to improve adherence.

## Introduction

Because of the extensive use of highly active antiretroviral therapy (HAART), human immunodeficiency virus (HIV) infection has become a chronic disease; therefore, more children who acquire HIV perinatally reach their adolescence and adulthood. Access to care and adherence to HAART are key factors for an effective treatment for all patients with HIV infection (Mills et al., 2006; Murphy et al., 2005). Adherence to antiretroviral therapy with a prescribed medication intake threshold equal to or more than 95% was associated with complete viral suppression (Gross et al., 2006; Lima et al., 2008, 2009), while a suboptimal adherence rate results in lower HAART plasma concentrations, inducing drug resistance to one or more of the given drugs and increasing the possibility of cross-resistance to drugs of the same class (Flynn et al., 2004; Howard et al., 2002; Murphy et al., 2005; Paterson et al., 2000).

In Panamá, by December 2012, a total of 13,095 cumulative cases of AIDS had been identified in the overall population. Perinatal transmission accounts for 2.8% of all reported cases (Cumulative report of the AIDS situation, 1984–2012). In 2011, HIV was ranked eighth among the causes of death in Panama in the overall population (Ministry of Health, 2012), with a cumulative number of 8731 AIDS-related deaths and a high mortality rate of 67% (Cumulative report of the AIDS situation, 1984–2012). Currently, a total of 220 HIV/AIDS patients diagnosed perinatally are receiving healthcare services at two pediatric centers in Panama City. Today, 95 (43%) of them have reached late childhood and adolescent age (10–18 years old), which supports the benefits of HAART for the treatment of HIV infection as a chronic health condition (Antiretroviral Therapy Clinic, 2010).

Adolescents are a vulnerable population, with many physical and emotional changes that normally occur within this period. Many factors may contribute to jeopardizing their adherence to lifelong treatment, resulting in frequently missed appointments and failure in refilling prescriptions on schedule (Porth, Suzuki, Gillespie, Kasedde, & Idele, 2014). In previous studies, a poor adherence rate of less than 50% was found among children and adolescents and/or caretakers, measured by self-reported medication intake, electronic monitoring devices, and/or drug pharmacy refill or count (Khan et al., 2009; Williams et al., 2006; Xochihua-Díaz, 2009).

All these factors emphasize the need to improve the understanding of the characteristics of our patient population and generate an appropriate treatment approach. This is the first study that explores the social and psychosocial factors associated with adherence among adolescents with perinatal HIV infection in Panama from a gender perspective.

## Methods

### Study design and population

We conducted a cross-sectional observational study of adolescents with HIV-1 infection from two main pediatric hospitals in Panama City, using a questionnaire to interview adolescent patients and caregivers (informants). Data were collected between 19 July 2013 and 11 November 2013.

Patients were eligible for the study if they were aged between 12 and 18 years, had a history of perinatal transmission of HIV-1, were undergoing antiretroviral therapy for at least one year, and attended their follow-up visits every 3 months. Patients were excluded if they were living outside one of the three central provinces of Panama (Panama, Panama Oeste, and Colon), which represent areas of large urban concentrations with access to the two third-level pediatric hospitals in which this study was conducted. Before the study started, patients expressed their willingness to participate, and each legal guardian provided written informed consent. This study was conducted in accordance with the principles of the Declaration of Helsinki, the World Health Organization Ethical Guidelines for Biomedical Research Involving Human Subjects, and the National Commission of Bioethics of Panama. The research plan was evaluated and approved by the local ethics committee of both participating hospitals (Pediatric Hospital “Dr. Jose Renan Esquivel” and Social Security Pediatric Hospital “Dr. Arnulfo Arias”, Panama City, Panama) and from “Instituto Conmemorativo Gorgas de Estudios de la Salud – Panama Health Ministry”. Previous to the interview, informed consent was signed by the legal guardian of each patient.

### Questionnaire

A questionnaire, developed for the study, based on previous studies, with a total of 119 questions, was applied by a social worker at the patients’ homes and was completed by both the informants (Questions 1–26) and patients (Questions 27–118). The last question (Question 119) regarding biological components was completed by the patients’ doctors.

The questionnaire was used to collect patient demographic information (e.g., gender, age, province address), explore social factors (e.g., living conditions, family members, meals, and education), and psychophysical well-being (e.g., activities of leisure, happiness, person most trusted by the patient, frequency of illness, sexual activity, contraception knowledge, and HIV information). Adherence to treatment was evaluated by asking the patients if they had missed any dose during the 7 days prior to the interview; and if so, they were asked to provide reasons for missing a treatment dose and any action plan to get the medication. The ability of patients to recall their medication and the methods used to remember to take their medications were also assessed and then verified by the patients’ medical records. Additionally, patients were asked about the presence of side effects to antiretroviral drugs. The biological components included viral load for the last three months and CD4 cell counts.

### Statistical analyses

Sample size calculations were based on a model recommended by the University of Florida, Institute of Food and Agricultural Sciences extension (IFAS) in which the sample is obtained through the extrapolation of previously established tables to achieve a statistical <0.05 significance. Statistical analyses were performed to investigate differences by gender. The type of analysis used depended on the type of data generated by the responses to the questionnaire. Quantitative tests were used to analyze differences by gender including *t*-tests, chi-square test, and Fisher chi-square test, while Pearson *r* and Spearman Rho tests were used for correlations. Qualitative analyses were done by categorizing when possible and then generating counts and percentages. A *p*-value <.05 was considered statistically significant.

## Results

### Study population

From a total of 95 adolescents with perinatal HIV-1 infection, 38 adolescents were randomly selected and included in this study. The demographic characteristics of the study population are shown in Table 1. Twenty patients (53%) were female and 18 (47%) were male, with a median age of 14 (range, 12–18) years. There was no statistically significant difference in the mean age of female and male patients (15.1 years and 14.5 years, respectively; *p* = .39).

**Table 1.** Demographic characteristics of the study population.

Characteristics	Value
Patients, *N*	38
Female, *n* (%)	20 (53)
Age, years, median (range)	14 (12–18)
Geographic distribution^a^, *n* (%)	
Province of Panama	25 (66)
Panama City	18 (47)
San Miguelito	6 (16)
Chepo	1 (3)
Province of Panama Oeste	10 (26)
Arraiján	6 (16)
Chame	2 (5)
La Chorrera	2 (5)
Province of Colon	3 (8)
Colon	3 (8)

^a^Provinces and districts.

There were 39 informants (both grandparents served as informants for one participant), with a median age of 43 years (18–77 years), 32 (82%) of them were females. All but two were family members: usually, the mother, aunt, or grandmother. Of the eight male informants, six (79%) cared for a male adolescent participating in the study.

### Social factors

#### Living conditions

The majority (*n* = 25; 66%) of the adolescents lived in the Province of Panama (*n* = 25), while the remaining adolescents lived in the provinces of Panama Oeste (*n* = 10; 26%) and Colon (*n* = 3; 8%).

#### Family

Both parents of only six adolescents (16%) were alive, and both parents of 12 adolescents (32%) were dead, while 17 fathers (45%) and 15 mothers (39%) were reported alive. Slightly over half of the adolescents (55%) lived with at least one parent and two to four family members.

Most of the patients (78%) were under the care of a female adult; in 60% of the cases, the adolescents named their grandmothers, mothers, or aunts as their caregivers. Twelve adolescents (32%) named more than one family member as their caregivers. Almost all of the adolescents said that they were treated well (90%) and were happy (87%) in their homes. When asked why they were happy, they said that they felt loved and supported by their family and their needs were met.

In 2013, the minimum monthly income for a four-member family in Panama was 319.09 USD, which covered the basic food basket (Ministry of Economy and Finance – Panama, 2013). Financially, 44% of the families lived on less than 399.00 USD per month, 42% lived on 400.00–599.00, and the remaining (14%) responded more than 600.00 USD. The main financial provider was reported to be one or both parents in 13 cases (34%).

#### Meals

The majority of the patients (79%; 16 female and 14 male) responded that they had three meals per day, seven patients (18%; 4 male and 3 female) had two meals per day, and only one patient responded that she ate only once a day.

#### Education

Thirty-two (84%) patients went to school, most of them to public schools. Of the six dropouts (four male and two female), five planned to go back to school. Almost all (95%) were treated well by their teachers and peers because of their personality and behavior. More than half of them (*n* = 27; 58%) went to their regular grade. Slightly more than half (*n* = 21; 55%) responded that they had everything they needed for school, and 35% obtained financial help (scholarship). Most patients had their school expenses paid by a female relative (35/55 responses; 66%), and in more than a third of the cases, several relatives provided support (13/38 patients; 35%). Thirty-three patients (87%) had plans for their future and named 24 different professional career choices.

### Psychophysical well-being

In their daily life, the patients behaved like typical teenagers. In their spare time, most of them reported watching TV, studying, listening to music/dancing, playing, playing videogames, doing sports, and hanging out with their friends.

They considered themselves happy, but more than half were worried about their future (*n* = 23; 61%), academic performance (*n* = 21; 55%), their illness (*n* = 20; 53%), and death (*n* = 19; 50%) with no difference by gender. When asked about whom they trusted most when they were sad or worried, four boys and three girls chose more than one person; “grandmother” was chosen more often by boys, and “friends” was chosen more often by girls. Girls were equally likely to seek a family member (*n* = 11) or a nonfamily member (*n* = 12), whereas boys were more than twice as likely to seek a family member (*n* = 18) than a nonfamily member (*n* = 7) (*X* ^2^ = 2.92; *p* = .09).

Most of the patients reported that they got along well with all of their family, but for those who had problems, fathers (*n* = 5) and brothers (*n* = 4) were considered the usual source of friction, with no significant difference in the responses according to gender.

#### Health

Eighteen patients reported becoming ill once a year (47%), and six patients reported becoming ill once every 3 months (16%). The most prevalent illness was “flu and cough” (71%) along with fever (34%) and colds (21%).

When the patients did get sick, 76% were taken to the doctor. Most of the patients (*n* = 31; 82%) saw a doctor at least every three months. Almost all patients (*n* = 35; 92%) visited a specialist in infectious diseases and many visited a psychologist (*n* = 25; 66%) and/or an odontologist (*n* = 20; 53%). A higher proportion of boys were taken to the psychologist (difference by gender, *X* ^2^ = 4.68, *p* = .03).

Almost all patients (*n* = 31; 82%) said that they knew what sexual relations were. Only four patients reported having sex; three were girls who said that they had heterosexual, vaginal sex with adults, and one boy said that he had sex with a minor female. None said that they had sex against their will. When asked about contraception, two patients reported always using condoms, and the other two responded “sometimes”. One girl said she received injections.

When the whole group was asked about knowledge of contraceptives, and specifically about condoms, 31 patients (82%) knew what they are. All but one patient knew about HIV-1, and 20 patients (53%) also knew about sexually transmitted diseases, although none of them thought that they had one.

### Adherence to antiretroviral treatment

Almost all patients (*n* = 36; 95%) gave a rational medical reason for taking their medications, such as “to keep the virus count low” and “it keeps me from getting sick”.

Male patients gave more fatalistic answers related to death, such as “to stay alive” and “not to die”, while female patients gave more responses about “keeping viral load low” and “not get sick” (*p* = .04).

Most of the patients (*n* = 36; 95%) responded affirmatively when asked if they knew what medications they were taking; however, when this information was matched with their medical records, the medication was recalled correctly by only 17 patients (45%). Although more female than male patients remembered the name of their medications, the difference between gender did not reach statistical significance (50% vs. 39%, respectively; *p* = .13).

Regarding the question about which methods the adolescents used to remember to take their medications, most of them (*n* = 30; 79%) responded that they relied on a family or nonfamily member. Additionally, only four patients named more than one method. Table 2 shows a detailed description of the different methods mentioned. When patients were asked specifically if someone at home supervised their medication intake, 86% of the patients responded that they relied on an adult. The same question asked to their informants showed consistency (92%) and good correlation (*r* = 0.75; *p *< .001) which indicated questionnaire reliability. Mostly female family members (25 female relatives vs. five male relatives) kept track of their medications. All patient responses regarding medicine intake supervision are described in Table 3.

**Table 2.** Antiretroviral treatment adherence reminder methods by gender.

Reminder method	Female	Male	Total^a^
Family member or nonfamily member^b^	12	18	30
Diary	4	2	6
Seven-day pillbox	2	3	5
None	3	2	5
Beep alert	2	2	4
Prescription labels on medicines	1	1	2
Beeping watch	2	0	2
Calendars	0	1	1
Cell phone notes	1	0	1
Total	27	29	56

Note: This table reflects patients’ responses when they were asked specifically about the methods they used to remember to take their medication.

^a^Four patients named more than one method.

^b^Including “Friends”.

**Table 3.** Patients’ responses on medication supervision.

Adult supervision	Female *N* = 19	Male *N* = 18	Total *N* = 37 (%)^a^
Positive responses	15	17	32 (86)
Negative responses	4	1	5 (14)
Who supervises			
Female relatives			25/39 (64)
Mother	4	5	9
Grandmother	1	7	8
Aunt	3	0	3
Sister	2	1	3
Stepmother	0	1	1
Stepsister	1	0	1
Male relatives			5/39 (13)
Grandfather	0	2	2
Cousin	1	0	1
Uncle	1	0	1
Guardian	0	1	1
Not specified	4	5	9/39 (23)

^a^There was one missing answer from a female patient. Six patients gave more than one response.

During the week prior to the interview, only 12 patients (32%) reported that they had not missed a dose, while 26 (68%) reported that they had missed at least one dose. The most common reason for missing a dose given by almost 70% of the respondents was “I forgot”. However, female patients gave more than twice as many excuses for not taking their medications than the male patients (*p* = .02).

Regarding adverse effects related to antiretroviral drugs, half of the patients reported no side effects. The other half mentioned more frequently nausea, abdominal pain, vomiting, and dizziness. Although more female (*n* = 12) than male (*n* = 7) patients reported side effects, this difference was not statistically significant (*p* = .19).

More than half (24/38 patients; 63%) of the patients said that they would remember to take their medication with them if they knew they would not be home for the next scheduled dose, and slightly more than half described an action plan of what they would do if they ran out of medication (55%). The action plan described more often was to go to the hospital to get more medication or tell an adult who would get the medication at the hospital or clinic. More male patients than female patients were likely to describe an action plan (male = 13 vs. female = 8; *p* = .05).

### Biological components

Only 26% of all patients (female = 6, male = 4) had an undetectable viral load (below 50 copies/mL) and 55% (female = 12; male = 9) 60% (female = 14; male = 9) showed no CD4 suppression (CD4 count above 500 cells/mm^3^).

## Discussion

Poor adherence to therapy is a particular concern in the management of all patients with chronic diseases, including the adolescent population (Datye, Moore, Russell, & Jaser, 2015; Srof, Taboas, & Velsor-Friedrich, 2012). Low adherence rates (<50%) to HAART on HIV-infected adolescent populations are frequently encountered (Murphy et al., 2003; Murphy, Wilson, Durako, Muenz, & Belzer, 2001). Previous studies on children with HIV infection have demonstrated an association between the reported antiretroviral adherence and the virologic response (Farley et al., 2008; Van Dyke et al., 2002).

Within our Panamanian adolescent cohort with perinatal HIV infection, adherence was found to be poor. Only one-third responded not missing a dose during a one-week period and even less had viral/immunological control. However, most adolescents responded that they felt happy at home, gave rational medical reasons for taking their medication, that they visited their doctors regularly. Almost half of them reported “getting sick” once a year, and no serious adverse effects were reported. This entire scenario shows that treatment adherence among the adolescent population is a real challenge and emphasizes the need to generate adolescent-sensitive approaches to facilitate their healthy transition into adulthood.

Among Latin adults with HIV infection, the most commonly found barriers were depression, simply forgetting, and sleeping through a dose (Murphy, Roberts, Hoffman, Molina, & Lu, 2003). In line with our study, Buchanan et al. found that the most common reason for missing a dose among children/youth with perinatally acquired HIV reported by patients and caregivers was forgetting to take the prescribed medications (Buchanan et al., 2012). The present study helps us to understand the actual scenario of our adolescent patients with a history of perinatal HIV transmission who are currently under medical care and receiving treatment. Their anti-HIV medication adherence was far below our expectations. Our findings highlight the need for the development of support strategies to accelerate improvement and promote equity in the adolescent population. It is imperative to identify which barriers impede medication adherence in each patient, including frequency of dosing, adverse events, adult/s responsible for intake supervision, psychological status, behavioral characteristics, and social context. Educational interventions on adolescents are important because many behavior patterns (interpersonal relationships, sexual behavior, and alcohol and drug use) are acquired during this stage and last a lifetime. Adolescents could be receptive to new ideas, with curiosity and interest in learning new habits (Funck-Brentano et al., 2005; Marciani & Moreno, 2001; Mosquera & Mateus, 2003). In the present study, only four of our adolescent patients named more than one method to remind them of taking their medications, and the majority still depended on only one family member – mainly female relatives such as the mother, grandmother, sister, or aunt. Regarding the gender perspective, female patients had more excuses for not taking their medication, while male patients were more likely to describe an action plan for what to do if they ran out of medication. No other gender differences were found between patients. Existing evidence supports the concept that more than one intervention to enhance adherence should be considered after evaluation of patients’ daily routine, such as cognitive-behavioral therapy, health education, treatment supporters, directly observed therapy, and reminder devices (e.g., alarms, mobile phone text messages, pillboxes) to help them find more autonomy (Chaiyachati et al., 2014; Murphy et al., 2005; Panel on Antiretroviral Guidelines for Adults and Adolescents, 2015). The impact and usefulness of the added interventions in each patient performance should be subsequently evaluated. Moreover, for maintaining adherence and patient retention, the type of interventions used may need to change as children grow. This was evidenced by Merzel et al., who evaluated perinatally infected children with HIV and observed that the oldest ones presented more regimen fatigue and resistance to taking their medications (Merzel, VanDevanter, & Irvine, 2008).

Relevant health actions for prevention and treatment programs focused on the pediatric population with HIV are required to counterbalance the disadvantageous health situation in low-resource countries compared with high-resource countries (Hazra, Siberry, & Mofenson, 2010). Civil society interventions targeting adolescent populations have resulted in positive experiences. Although policies and programs to ensure the respect of adolescent patient rights are more frequently implemented in urban areas, these are scarce in urban areas where the access to health services and prevention programs is restricted (OPS/OMS, 2007).

During the study, although a small number of patients referred being sexually active, only half of them regularly use protection during their sexual intercourses. Moreover, none of the patients thought that they had a sexually transmissible disease. This unexpected finding reinforces the need to provide them with more education about HIV and other sexually transmitted diseases. Additionally, further counseling on family planning, including contraception methods and safer sex techniques is paramount to prevent secondary HIV transmission (Panel on Antiretroviral Guidelines for Adults and Adolescents, 2015). Furthermore, among a cohort of Spanish adolescents with vertically transmitted HIV infections studied by García-Navarro et al., more efforts on health education were evidently required although a high percentage of their patients knew their diagnosis, but only 30% understood the disease and six unexpected pregnancies were reported (Garcia-Navarro et al., 2014).

The present study has some limitations, including the small sample size and the inherent limitations of a questionnaire-based study, where the results rely mostly on the accuracy of patient responses. Furthermore, the gathered information is only from regularly active patients in three central provinces of Panama, and the scenario could be worse in other rural areas where access to treatment is even more difficult for all patients.

## Conclusions

Most adolescents still depend on their female relatives to remind them of taking their medications, and no remarkable differences in factors associated with adherence were found in regard to the gender perspective. Improved adherence to antiretroviral therapy in adolescents is required in Panama. To address this issue, educational programs that involve not only the patients but also their family members are warranted to improve the adolescents’ independence and adherence-related habits.
